# A study on the equity of self-rated health of older adults at the family level

**DOI:** 10.1186/s12939-023-01895-6

**Published:** 2023-04-25

**Authors:** Weicun Ren, Yiqing Xing, Clifford Silver Tarimo, Ruibo He, Zhang Liang

**Affiliations:** 1 School of Political Science and Public Administration, Wuhan University, Wuhan, China; 2 College of Public Health, Zhengzhou University, Zhengzhou, China; 3 Dares Salaam Institute of Technology, Department of Science and Laboratory Technology, Dares Salaam, Tanzania

**Keywords:** Older adult, Self-rated health, Family, Equity, Coupling coordination

## Abstract

**Background:**

The self-rated health of older adults (SHOA) plays an important role in enhancing their medical service utilization and quality of life. However, the determinants and magnitude variations in SHOA at the family level (SHOAFL) remain unknown. The purpose of this study was to assess the status and equitable level of SHOAFL in China, as well as to analyze the influencing factors and the precise nature and scope of their impacts.

**Methods:**

This study analyzed the data from the "Chinese residents' health service needs survey in the New Era", and included a total of 1413 families with older adults. The status and influencing factors of SHOAFL were analyzed using mean comparison and Logistic regression (LR) models. The Concentration Index method was used to explore the equity of the distribution of SHOAFL. The relationship between differences in personal characteristics among family members and differences in SHOA was determined by the method of Coupling Coordination Degree (CCD).

**Results:**

The total score of SHOAFL was 66.36 ± 15.47, and LR results revealed that the factors with a significant impact on SHOAFL were number of people living in family, distance to the nearest medical service institution, travel time to the nearest medical service institution, annual family income, yearly family medical and health expenditures, average age, and residence (all *P* < 0.05). The Concentration index of SHOAFL ranged from -0.0315 to 0.0560. CCD of the differences between SHOA and medical insurance and smoking status were 0.9534 and 0.7132, respectively.

**Conclusion:**

The SHOAFL was found to be generally but more inclined towards urban families with high incomes and a short time to medical service institution. The observed disparities in SHOA among family members were mostly attributable to differences in health insurance and pre-retirement occupations. The status and equality of SHOAFL may be improved if policymakers prioritize making services more accessible to older rural residents with low incomes. Concurrently, reducing the existing discrepancy in health insurance coverage between older couples may also enhance their health.

## Introduction

At present and in the foreseeable future, the improvement of medical care and the reduction of mortality due to infectious diseases have made population aging a global trend [1]. As of 2020, there were 264 million old people aged 60 years and above in China, accounting for 18.70% of the total population, and 190 million old people aged 65 years and above, accounting for 13.50% of the total population [2]. Hubei, Guizhou and Guangzhou provinces are the most populous provinces in central and southern China, and newly industrialized and transitioning provinces. In 2020, the total number of older adults over the age of 65 in this three provinces exceeded 23 million, and the dependency coefficient of older adults was between 11.82% and 21.11% [3]. With the increase in the life expectancy of older adults, the physical state of older adults varies, and the functions of various organs and bodily functions decline, so making health a key issue in old age [4, 5]. In 2015, the World Health Organization (WHO) formally defined healthy ageing as "the process of developing and maintaining the functions required for healthy life in old age" [6]. The State Council of China issued the "Healthy China 2030" Plan Outline in 2016, which incorporated healthy aging into the country's long-term development plan [7].

The self-rated health status, also known as self-perceived health, refers to an individual's subjective evaluation and expectation of their health status, which can accurately reflect the overall state of their physical, psychological and social aspects [8]. The tool is frequently used to estimate potential functional capacities in older adults based on self-reported health status [9, 10]. The self-rated health of older adults (SHOA) is directly related to their personal well-being and health resource utilization [11]. Studies have shown that the single-item rating of SHOA is a strong predictor of future morbidity and mortality, and this variable has been recommended as an indicator in international comparative studies [12]. Lu ZF et al. [13] found that the SHOA in rural western China was not ideal, while An RJ et al. [14] found that improving the SHOA can effectively prevent the occurrence of depression based on a nationwide study.

Studies have shown that SHOA is associated with social determinants, such as intergenerational support during the life course and later in life, access to and utilization of medical services, place of residence, and economic status [15–17]. Shi YF found that intergenerational support has a significant positive impact on SHOA, and indirectly affects SHOA by affecting their mental health [18]. And Mackenbach et al. discovered the Nordic Paradox, in which, while having some of the most comprehensive social safety programs, Scandinavian nations tend to have a rather high gap in SHOA between socioeconomic classes [19]. According to a Mexican health care survey, a person's self-rating of his health is moderate or poor if he has a high frequency of doctor visits or a large desire for medical treatment [20]. In addition, Li XR et al. concluded that older adults who were unable to travel from their place of residence to the nearest medical institution within 15 min had poorer self-rated health outcomes [21]. Living predominantly in rural or urban areas has also been shown to be associated with SHOA [15].

In addition, various studies have shown that SHOA also depends on a variety of personal characteristics such as age, pre-retirement occupation, education level, smoking, and exercise [22–24]. In terms of age, younger older adults generally had better self-rated status than older adults [25, 26]. In China, Yang YC et al. reported that income sources such as pre-retirement occupations can impact SHOA [27]. Using 47 years of repeated cross-sectional data to predict trends in SHOA, Schellekens J and colleagues discovered that those with higher education showed a higher increase in self-rated health than those at lower education levels [28]. By evaluating the effect of lifestyle choices on SHOA, Shield M discovered that heavy smoking, inability to ensure regular exercise, and obesity can decrease self-rated health [29]. When universal coverage for basic medical services was attained, it was also shown that reasonably advanced medical insurance improved SHOA [18, 30].

To sum up, there have been many studies on SHOA, but most of these studies focus on investigating and analyzing the health status, willingness and influencing factors of older adults. Limited studies have explored the families of elderly people, focusing mostly on child care, spiritual support, and other health-related outcomes, with little attention paid to the allocation and equity of SHOA within the family unit. In this regard, the novelty of this study compared with existing studies is that it takes an older adult’s family as the basic unit to objectively and statistically evaluate the factors affecting SHOA at the family level (SHOAFL) and the equity of its overall distribution. It also discussed the interaction between differences in personal characteristics of older adults couple and differences in their self-rated health status.

To help develop effective older adult care strategies and improve the efficiency of older adults’ healthcare services from a holistic and sustainable development perspective, the status and influencing factors of SHOAFL were analyzed using mean comparison and Logistic regression (LR) methods in this study. The Concentration Index method was used to explore the equity of the distribution of SHOAFL. The relationship between differences in personal characteristics among family members and differences in SHOA was determined by Coupling Coordination Degree (CCD) method.

## Methods

### Data sources

The data used in this study come from the "Chinese residents' health service needs survey in the New Era" that was carried out in July–August 2018 [31–33]. The study was conducted within the context of a healthcare model in which inhabitants often receive specialized and high-quality medical care at large public hospitals located at the municipal level and above. Most citizens have easy access to county hospitals, township health facilities, and village health offices, which provide basic healthcare services. However, the referral model within the county, which is supposed to adhere to a village-town-county structure, is not always properly implemented, and residents have a great deal of choice about where to seek healthcare services.

The survey adopted a multi-stage stratified random sampling method for sample selection. In China's rural and urban areas, two counties and two districts (Futian District, Xiling District, Sinan County, and Dangyang County) were selected in the first stage based on representativeness and difference in population and economic status. In the second stage, in each county (district), 5 townships (streets) were selected according to the geographical distance from the medical institution, and 6 natural villages (communities) were selected according to the geographical distance from each township (street); In the third stage, a systematic random sampling method was used to select 40 families in each natural village (community), considering that some respondents may refuse, additional households were included in the sample, and at least 42 families were surveyed in each village (community) [31]. All sampled families were systematically selected from the resident register of the village committee (neighborhood committee), and all members of the sampled families were surveyed. Inclusion criteria: (1) Be on the resident register of the local village committee (neighborhood committee); (2) Agree to participate in the survey; (3) Know the basic content of residents' health services.

**Fig. 1:**
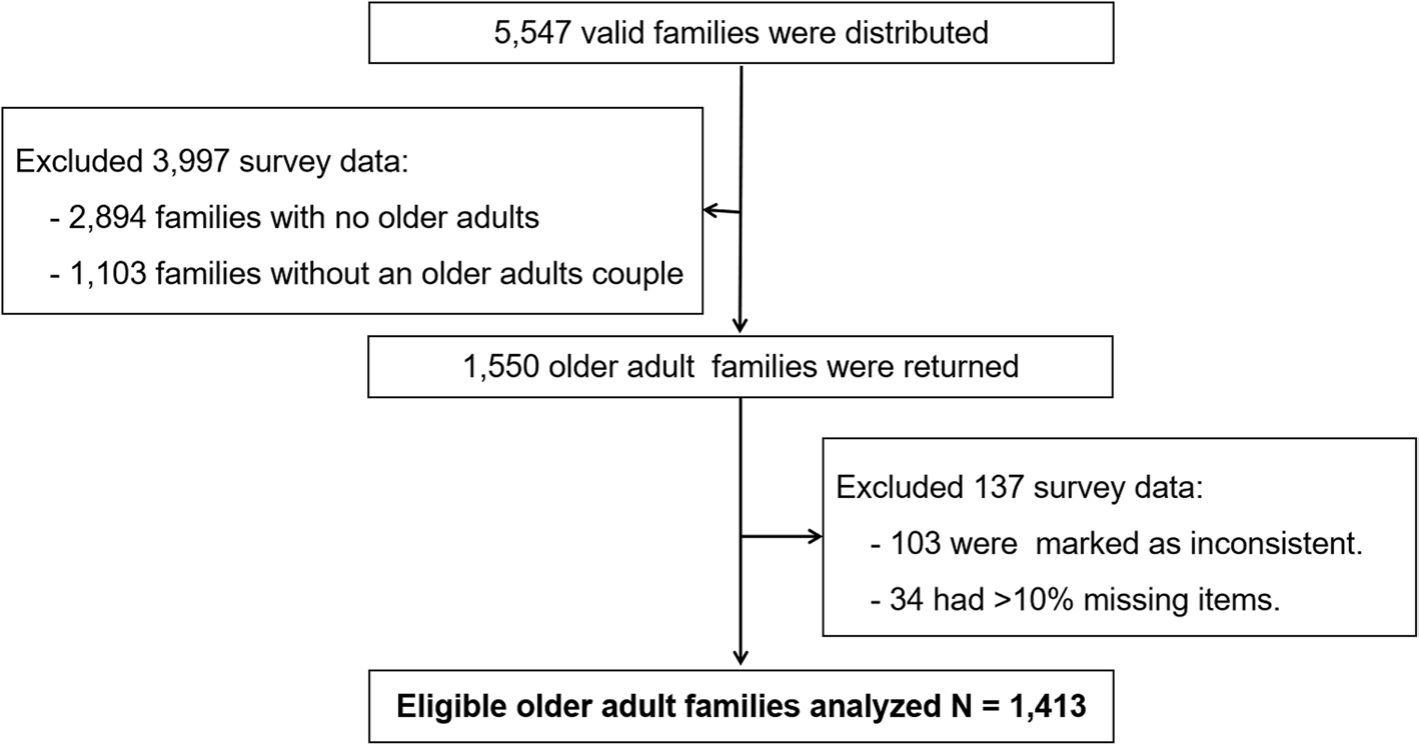
Schematic diagram for sample size determination

In the sample size calculation, the design effect was set at 2.5, with an allowable error at a significant level of 0.05 with the prevalence of chronic diseases in the population set at 21.34%. A minimum sample size of 3,600 participants in 30 villages (communities) per study center was established, and a total of 15,126 questionnaires were collected. This study selected older adult families in the sample (each family contains one male and one female older people aged 60 and above who were in a marital relationship) as study participants. Considering that there were no missing answers to key variables such as age and total family income, the final inclusion contained 1,413 families with a total of 2,826 older adults were analyzed (Fig. 1).

The survey was conducted by a group of undergraduate and postgraduate students majoring in health management, preventive medicine, et al. All interviewers already had relevant professional knowledge and received training. The survey was mainly conducted in a question-and-answer format. And investigators completed face-to-face interviews and filled out the questionnaires on the spot. The survey respondents were informed of the relevant circumstances of the survey in advance and agreed to participate in the survey. Questionnaires were checked and entered on the same day after collection and reviewed by experienced supervisors. Questionnaires that did not match the facts or were missing were supplemented via telephone, and those that could not be supplemented or were still missing were deemed unqualified. After evaluation by the Ethics Committee of Tongji Medical College of Huazhong University of Science and Technology, the content and procedures of this study met the ethical requirements of international and national biomedical research (IORG number: IORG0003571).

### Evaluation variables

Based on the perspective of family-individual integration, this study used a questionnaire for “Chinese residents' health service needs survey in the New Era” to investigate. The questionnaire includes five parts: basic family information, personal demographic background, self-rated health status, hygiene habits and medical care utilization, and has good reliability and validity [31, 32]. Indicators used in this study mainly include the basic information of the older adult family, the personal demographic background, self-rated health status and hygiene habits of older adults in the family. The main outcome variable was the SHOAFL of the respondents. Self-rated health of older adults was measured by the three-level European five-dimensional health scale (EQ-5D-3L) [34, 35], and the results of the visual analogue scale (VAS) ranging from 0 to 100 were used to reflect SHOA. The SHOAFL was represented by the average SHOA of older couples in the family. The meaning of variables were described in Table 1 [18, 36].

**Table 1:** The meaning and assigned value of indicators

Variable category	First-level indicators	Secondary indicators	Meaning	Assigned value
Independent variables	Basic family information	Number of family people	The total number of people living in the family	Number
		Medical service institution	The type of medical service institution closest to the family’s residence	1-5^a^
		Distance	The distance to the nearest medical service institution	Distance (KM)
		Travel time	The time it takes to get to the nearest medical service provider using the most commonly used means of transportation	Time (Minutes)
		Family income	Yearly total family income	Yuan/year
		Medical and health expenditure	Yearly family medical and health expenditure	Yuan/year
		Average age of older couples	The average age of older couples in the family, residence	Age
		Residence	Is the main residence of the family urban or rural	1-2^b^
	Personal demographic background	Age	The age of the older adults	Age
		Education level	Education level of the older adults	1-5^c^
		Pre-retirement occupation	Occupations in which older individuals spend the majority of their time prior to retirement	1-5^d^
		Medical insurance	The main type of medical insurance that older adults possess	1-2^e^
		Means of communication	The communication tools and methods that older adults mainly use in their daily life	1-4^f^
	Health habits	Smoking status	Whether smoking (smoking refers to those who have smoked continuously or accumulatively for 6 months or more)	1-3^j^
		Drinking status	Whether the participant consumes alcohol (drinking refers to consuming alcoholic beverage at least once a week for six months or more)	1-3^j^
		Exercise status	Average weekly physical activity in the past 30 days	0-7^ h^
Outcome variable	Self-rated health	Mobility	Difficulty in completing physical actions independently	1-3^i^
		Self-care	Difficulty in completing self-care (washing, dressing, going to the bathroom, et al.)	1-3^i^
		Usual activities	The degree of difficulty in engaging in ordinary activities (work, reading, or doing housework)	1-3^i^
		Pain/discomfort	The degree of physical pain or discomfort	1-3^i^
		Anxiety/depression	The degree of anxiety or depression	1-3^i^

^a^1-5refers to pharmacy, private hospitals and private clinics, community health service station/village clinic/outpatient department, community health service center /township health center, county-level and above public medical and health institutions; ^b^0 means "Rural", 1 means "Urban"; ^c^1-6 indicates that the education level is illiterate, primary school, junior high school, high school or technical secondary school, junior college and above; ^d^1-5 refers to agricultural production personnel or unemployed, commercial/service industry personnel, workers, professional and technical personnel, and civil servants; ^e^1-2 represents basic medical insurance for urban and rural residents, basic medical insurance for urban employees, commercial medical insurance, et al.; ^f^1-4 represents smartphone, non-smartphone, landline, verbal or other; ^j^1 means smoking or drinking, 2 means quitting smoking or drinking, 3 means no smoking or drinking; ^h^0 means not exercising, 1–6 means the average frequency of physical exercises per week was 1 to 6 times, and 7 means the average number of physical exercises per week was 7 times or more; ^i^1 means that the function or ability of older adults was impaired or extremely low, and 2 means that the function or ability was normal

### Concentration index

The Concentration Index is one of the commonly used methods to measure equity, and its results indicate the degree of concentration of the distribution of health or health status among different geographical or population level [37]. Continuous and discontinuous data necessitate distinct calculation methods for concentration indices. The geometric technique is specifically applied to continuous data, while the covariance method is applicable to discrete data [38]. Considering that the SHOAFL was set as continuous data in this study, the geometric method was used to calculate the Concentration Index(*G*) of SHOAFL:

$$\text{G}=1-\sum\limits^{1412}_{i=0}\left(x_{i+1}-x_i\right)\left(y_{i+1}+y_i\right)$$

Among them, x_i_ is the cumulative percentage of the number of older adult families; *y*_*i*_ is the cumulative percentage of SHOAFL. The concentration index ranges from -1 to 1, and the closer the absolute value of the concentration index is to 0, the more equity it is. If the value of the concentration index is negative, indicating that the SHOAFL is concentrated in the families with a small number of people living in the family, a short time to the nearest medical service institution, and low income and medical and health expenditure; the concentration index is positive indicating the SHOAFL concentration in opposite levels of older adult families.

### Coupling Coordination Degree (CCD) model

The role of the CCD model is to quantify the degree of interaction between two or more systems or between various elements within them, which not only reflects whether each system has an interaction relationship, but also reflects the degree of mutual influence and promotion between systems [39]. The formula for calculating the CCD of two systems is as follows:

$$\begin{array}{l}y_{1i}=\frac{x_{1i}-x_\text{1min}}{x_\text{1max}-x_\text{1min}},\,y_{2i}=\frac{x_{2i}-x_\text{2min}}{x_\text{2max}-x_\text{2min}},\\d_j=1+\frac{\sum\limits^{1413}_{i=1}y_{j_{i}}\text{ln}\,y_{j_{i}}}{\text{ln1413}},\,w_j=\frac{d_j}{\sum\limits^{2}_{j=1}d_j}\\\text{U}_1=\frac{\sum\limits^{1413}_{i=1}y_{1i}}{1413},\,U_2=\frac{\sum\limits^{1413}_{i=1}y_{2i}}{1413}\\\text{C}=\frac{\sqrt{{U}_1{U}_2}}{\frac{U_1+U_2}{2}},\,T=w_1\text{U}_1+w_2\text{U}_2\\\text{D}=\sqrt{C{\times}T}\end{array}$$

Among them, *y*_*1i,*_ *y*_*2i,*_ *x*_*1i,*_ *x*_*2i,*_ *x*_*1max,*_ *x*_*2max,*_ *x*_*1min,*_ *x*_*2min*_ represent the standardized value, original value, maximum value and minimum value of differences in SHOA and differences in personal characteristics among family members (*i* = 1, 2, 3, ..., 1413); *dj* and *wj* represent the information utility value and weight of differences in SHOA and differences in personal characteristics, respectively (*j* = 1, 2). *U*_*1*_ and *U*_*2*_ represent the comprehensive evaluation values of differences in SHOA and differences in personal characteristics, respectively. *C* is the degree of coupling between the two systems, *T* represents the total evaluation value of differences in SHOA and differences in personal characteristics. *D* represents CCD, CCD ∈ [0, 1]. The closer the CCD is to 1, the stronger the coupling and coordination between the two systems [40].

### Statistical analysis

Mean comparison and Logistic regression (LR) methods were used to analyze the SHOAFL and its influencing factors. The equity of the distribution of SHOAFL was explored using the Concentration Index method. The relationship between differences in personal characteristics among family members and differences in SHOA was determined using the CCD. *P* value < 0.05 was considered to be statistically significant. Data enter was done using Epidata 3.1 software, and statistical analysis was performed using Excel 2019 and SPSS 20.0 software.

## Results

### Characteristics of the older adult families

The basic characteristics of the older adult families included in this study were described in Table 2. Among the families participating in the survey, 67.52% of the families have a total number of people living in a family of 2, rural and urban families account for 56.48% and 43.52%, respectively, and the average age of older adults in the family was less than 65 years old and the families with the average age of 80 years or older account for 31.99% and 4.81%, respectively. More than half of the families were less than 1 KM away from the nearest health service institution. 16.70% of families had an annual total income of less than 10,000 yuan, and 19.25% of families had annual household medical and health expenditures of less than 1,000 yuan. At the same time, the analysis found that there were significant differences in SHOAFL of the families with different total number of people living in the family, medical service institution closest to home, distance to the nearest medical service institution, time to the nearest medical service institution, total family income, yearly family medical and health expenditure, average age of older adult couples in the family, and family residence (*P* < 0.05).

**Table 2:** Characteristics of the families (*N* = 1,413)

Index	Family (Number (%))	Score^a^ ($\overline{x} \pm s$*)*	*t/F*	*P*
**Family population**^**b**^** (number)**				
Two	954 (67.52)	66.47 ± 15.44	3.205	0.012
Three	206 (14.58)	67.28 ± 14.75		
Four	117 (8.28)	65.18 ± 16.64		
Five	101 (7.14)	67.68 ± 14.63		
Six and above	35 (2.48)	57.87 ± 16.72		
**Medical service institution closest to home**				
Community health service station/village clinic/outpatient department	815 (57.68)	61.21 ± 15.46	63.840	< 0.001
Community health service center /township health center	153 (10.83)	71.60 ± 14.89		
County-level and above public medical and health institutions	84 (5.94)	72.90 ± 11.86		
Pharmacy	331 (23.43)	74.06 ± 11.59		
Private hospitals, private clinics, et al	30(2.12)	76.12 ± 7.62		
**Distance to the nearest medical service institution (KM)**				
Less than 1	902 (63.84)	68.59 ± 14.89	25.929	< 0.001
1–2	343 (24.27)	64.64 ± 15.30		
2–3	107 (7.57)	58.49 ± 15.97		
3 and above	61 (4.32)	56.81 ± 14.87		
**Time to the nearest medical service institution (Minutes)**				
0–5	198 (14.01)	68.09 ± 14.58	34.856	< 0.001
5–10	649 (45.93)	69.89 ± 14.52		
10–15	294 (20.81)	65.25 ± 15.23		
15–20	83 (5.87)	61.78 ± 14.00		
> 20	189 (13.38)	56.16 ± 15.48		
**Total family income (yuan/year)**				
Less than 10,000	236 (16.70)	55.17 ± 15.24	82.150	< 0.001
10,000–29,999	311 (22.01)	61.67 ± 14.15		
30,000–79,999	521 (36.87)	68.77 ± 14.13		
80,000–149,999	241 (17.06)	73.37 ± 13.22		
150,000 and above	104 (7.36)	77.41 ± 10.58		
**Yearly family medical and health expenditure (yuan/year)**				
Less than 1000	272 (19.25)	69.24 ± 15.90	3.445	0.008
1000–2999	347 (24.56)	66.04 ± 15.80		
3000–7999	394 (27.88)	66.14 ± 15.39		
8000–14,999	191 (13.52)	64.41 ± 15.05		
15,000 and above	209 (14.79)	65.31 ± 14.48		
**Average age**^**c**^** (years)**				
< 65	452 (31.99)	68.43 ± 14.77	3.168	0.013
65–70	461 (32.62)	64.99 ± 15.72		
70–75	293 (20.74)	65.90 ± 16.06		
75–80	139 (9.84)	65.54 ± 15.21		
≥ 80	68 (4.81)	65.49 ± 15.33		
**Residence**				
Rural	798 (56.48)	60.42 ± 15.45	-18.940	< 0.001
Urban	615 (43.52)	74.06 ± 11.63		

^a^Score: The score of self-rated health of older adults at the family level (0–100); ^b^Family population: The total number of people living in the family; ^c^Average age: Average age of older couples in the family

### The SHOAFL

The survey results showed that the SHOAFL was 66.36±15.47, and the evaluation results for urban and rural families were 74.06±11.63 and 60.42±15.45, respectively. Specific to each evaluation dimension, the evaluation of self-care ability was relatively the highest (2.85±0.33), and the score of physical pain was only 2.59±0.46. See Table 3.

**Table 3:** The self-rated health of older adults at the family level (SHOAFL)

Variable	Total			Urban			Rural		
	Family -level	Male	Female	Family -level	Male	Female	Family -level	Male	Female
**Self-rated health status**	66.36 ± 15.47	66.99 ± 17.05	65.72 ± 17.35	74.06 ± 11.63	74.16 ± 12.57	74.31 ± 12.95	60.42 ± 15.45	61.52 ± 17.98	59.10 ± 17.42
Mobility	2.78 ± 0.36	2.78 ± 0.44	2.78 ± 0.44	2.87 ± 0.29	2.88 ± 0.36	2.87 ± 0.38	2.71 ± 0.39	2.70 ± 0.48	2.72 ± 0.47
Self-care	2.85 ± 0.33	2.85 ± 0.40	2.84 ± 0.41	2.92 ± 0.25	2.92 ± 0.30	2.91 ± 0.33	2.80 ± 0.37	2.80 ± 0.45	2.79 ± 0.45
Usual activities	2.77 ± 0.38	2.78 ± 0.46	2.77 ± 0.47	2.88 ± 0.29	2.89 ± 0.35	2.87 ± 0.39	2.69 ± 0.43	2.70 ± 0.52	2.69 ± 0.51
Pain/discomfort	2.59 ± 0.46	2.61 ± 0.53	2.57 ± 0.55	2.78 ± 0.37	2.77 ± 0.43	2.78 ± 0.44	2.44 ± 0.46	2.48 ± 0.56	2.40 ± 0.58
Anxiety/depression	2.84 ± 0.33	2.85 ± 0.37	2.83 ± 0.39	2.96 ± 0.17	2.96 ± 0.20	2.96 ± 0.19	2.74 ± 0.40	2.76 ± 0.43	2.73 ± 0.47

### Analysis on the influencing factors of SHOAFL

The LR method was used to analyze the influencing factors of family characteristics of SHOAFL. According to the univariate analysis and existing research results of old care services [17–19], the total number of people living in the family, medical service institution closest to home, distance to the nearest medical service institution, travel time to the nearest medical service institution, total family income, yearly family medical and health expenditure, average age of older couples in the family, and residence were included in the regression model as independent variables. In this study, the evaluation of SHOAFL was divided into two categories, and each SHOAFL's results were assigned a score of 0 or 1. Refer to Table 4 for the variable score assignment.

**Table 4:** Variables assignment for Logistic regression model

Variables	Assignment
Self-rated health	< 70 = 0, ≥ 70 = 1
Total number of people living in the family	Two = 1, Three = 2, Four = 3, Five = 4, Six and above = 5
Medical service institution closest to home	Community health service station/village clinic/outpatient department = 1, Community health service center/township health center = 2, County-level and above public medical and health institutions = 3, Pharmacy = 4, Private hospitals and private clinics, et al. = 5
Distance to the nearest medical service institution	Less than 1 KM = 1, 1–2 KM = 2, 2–3 KM = 3, 3 KM and above = 4
Time to the nearest medical service institution	0–5 min = 1, 5–10 min = 2, 10–15 min = 3, 15–20 min = 4, > 20 min = 5
Total family income	Less than 10,000 = 1, 10,000–29,999 = 2, 30,000–79,999 = 3, 80,000–149,999 = 4, 150,000 and above = 5
Yearly family medical and health expenditure	Less than 1000 = 1, 1000–2999 = 2, 3000–7999 = 3, 8000–14,999 = 4, 15000and above = 5
Average age of older couples in the family	< 65 = 1, 65–70 = 2, 70–75 = 3, 75–80 = 4, ≥ 80 = 5
Residence	Rural = 0, Urban = 1

The results of regression analysis showed that the SHOAFL was mainly affected by total number of people living in the family, medical service institution closest to home, time to the nearest medical service institution, total family income, yearly family medical and health expenditure, average age of older couples in the family and residence (*P* < 0.05). Among them, SHOAFL of urban older adult families was 2.738 times that of rural families, and SHOAFL of families whose time to the nearest medical service institution is 5–10 min was 1.848 times that of families whose time is more than 20 min (*OR* = 1.848, *95%CI* = 1.149 ~ 2.973). The SHOAFL with a total number of 2, 3, 4, and 5 people living in the family was 5.379, 4.925, 3.588, and 4.003 times that of families with a permanent population of 6 or more, respectively (*OR* = 5.379, *95%CI* = 1.999 ~ 14.473; *OR* = 4.925, *95%CI* = 1.778 ~ 13.643; *OR* = 3.588, *95%CI* = 1.253 ~ 10.273; *OR* = 4.003, *95%CI* = 1.390 ~ 11.531). The SHOAFL for families with a total income of less than 10,000 yuan, 1000–29,999 yuan, 30,000–79,999 yuan and 80,000–149,999 yuan is 0.101, 0.211, 0.314 and 0.516 times that for families with a total family income of 150,000 yuan and above, respectively. (*OR* = 0.101, *95%CI* = 0.049 ~ 0.210; *OR* = 0.211, *95%CI* = 0.106 ~ 0.420; *OR* = 0.314, *95%CI* = 0.171 ~ 0.578; *OR* = 0.516, *95%CI* = 0.273 ~ 0.978). The effect of distance to the nearest medical service institution on the SHOAFL was not statistically significant (*P* > 0.05). See Table 5.

**Table 5:** Logistic Regression (LR) results

Indicators	*β*	*SE*	*Wals*	*P*	*OR (95%CI)*
**Total number of people living in the family (Six and above)**	-	-	13.343	0.010	-
Two	1.683	0.505	11.102	0.001	5.379(1.999 ~ 14.473)
Three	1.594	0.520	9.405	0.002	4.925(1.778 ~ 13.643)
Four	1.278	0.537	5.668	0.017	3.588(1.253 ~ 10.273)
Five	1.387	0.54	6.602	0.010	4.003(1.390 ~ 11.531)
**Medical service institution closest to home (Private hospitals and private clinics, et al.)**	-	-	17.991	0.001	-
Community health service station/village clinic/outpatient department	-1.172	0.526	4.972	0.026	0.310(0.111 ~ 0.868)
Community health service center /township health center	-0.474	0.546	0.753	0.385	0.622(0.213 ~ 1.815)
County-level and above public medical and health institutions	-0.754	0.568	1.759	0.185	0.471(0.154 ~ 1.434)
Pharmacy	-0.458	0.524	0.764	0.382	0.632(0.226 ~ 1.767)
**Distance to the nearest medical service institution (3 KM and above)**	-	-	1.683	0.641	-
Less than 1 KM	0.443	0.367	1.455	0.228	1.557(0.758 ~ 3.195)
1–2 KM	0.462	0.365	1.600	0.206	1.587(0.776 ~ 3.247)
2–3 KM	0.456	0.400	1.296	0.255	1.577(0.720 ~ 3.458)
**Time to the nearest medical service institution (> 20 min)**	-	-	10.388	0.034	-
0–5 min	0.226	0.287	0.621	0.431	1.254(0.714 ~ 2.202)
5–10 min	0.614	0.242	6.414	0.011	1.848(1.149 ~ 2.973)
10–15 min	0.249	0.247	1.017	0.313	1.283(0.790 ~ 2.083)
15–20 min	0.344	0.318	1.168	0.280	1.410(0.756 ~ 2.629)
**Total family income (150,000 and above)**	-	-	48.539	< 0.001	-
Less than 10,000	-2.293	0.373	37.828	< 0.001	0.101(0.049 ~ 0.210)
10,000–29,999	-1.555	0.351	19.672	< 0.001	0.211(0.106 ~ 0.420)
30,000–79,999	-1.157	0.311	13.852	< 0.001	0.314(0.171 ~ 0.578)
80,000–149,999	-0.661	0.326	4.117	0.042	0.516(0.273 ~ 0.978)
**Yearly family medical and health expenditure (15000and above)**	-	-	54.303	< 0.001	-
Less than 1000	1.592	0.233	46.672	< 0.001	4.916(3.113 ~ 7.763)
1000–2999	0.980	0.216	20.609	< 0.001	2.665(1.745 ~ 4.068)
3000–7999	0.688	0.207	11.070	0.001	1.990(1.327 ~ 2.984)
8000–14,999	0.330	0.236	1.950	0.163	1.391(0.875 ~ 2.209)
**Average age of older couples in the family (≥ 80)**	-	-	22.824	< 0.001	-
< 65	0.938	0.312	9.027	0.003	2.554(1.385 ~ 4.708)
65–70	0.528	0.308	2.943	0.086	1.696(0.927 ~ 3.100)
70–75	0.410	0.315	1.695	0.193	1.507(0.813 ~ 2.793)
75–80	-0.066	0.343	0.037	0.848	0.936(0.478 ~ 1.834)
**Residence (Urban)**	1.007	0.206	23.919	< 0.001	2.738(1.828 ~ 4.099)
**Constant**	-3.011	0.947	10.100	0.001	0.049

### Equity analysis of distribution of SHOAFL based on Concentration index

According to the above analysis results of influencing factors, the total number of people living in the family, time to the nearest medical service institution, total family income, yearly family medical and health expenditure, average age of older couples in the family were ranked in descending order in this study. The calculation shows that the distribution of SHOAFL among families with different characteristics was basically equal (Concentration index = -0.0315~0.0560). The analysis results of rural and urban areas showed that the maximum concentration index appears in the distribution of SHOAFL based on total family income (Concentration index = 0.0368), and the absolute value of the concentration index of the distribution of SHOAFL in rural and urban areas calculated based on the total number of people living in the family was all less than 0.001. Specific to each dimension, the equity of distribution of pain/discomfort was relatively the worst (Concentration index = 0.0325) (Table 6).

**Table 6:** Concentration index of the self-rated health of older adults at the family level

Variables		Family population^a^	Time^b^	Total family income	Expenditure^c^	Average age^d^
**Self-rated health**	Total	-0.0023	-0.0315	0.0560	-0.0107	-0.0089
	Rural	-0.0004	-0.0275	0.0368	-0.0217	-0.0280
	Urban	0.0006	-0.0044	0.0168	-0.0262	-0.0177
Mobility	Total	-0.0006	-0.0134	0.0173	-0.0044	-0.0120
	Rural	-0.0009	-0.0155	0.0146	-0.0079	-0.0188
	Urban	0.0012	-0.0012	0.0048	-0.0079	-0.0131
Self-care	Total	0.0004	-0.0106	0.0128	-0.0048	-0.0094
	Rural	0.0002	-0.0126	0.0121	-0.0089	-0.0146
	Urban	0.0017	-0.0015	0.0028	-0.0054	-0.0095
Usual activities	Total	-0.0005	-0.0137	0.0185	-0.0053	-0.0140
	Rural	-0.0013	-0.0133	0.0148	-0.0101	-0.0232
	Urban	0.0024	-0.0027	0.0028	-0.0087	-0.0137
Pain/discomfort	Total	0.0007	-0.0201	0.0325	-0.0056	-0.0025
	Rural	-0.0002	-0.0133	0.0185	-0.0146	-0.0152
	Urban	0.0049	-0.0078	0.0061	-0.0143	-0.0083
Anxiety/depression	Total	-0.0053	-0.0114	0.0157	-0.0025	0.0004
	Rural	-0.0083	-0.0096	0.0080	-0.0104	-0.0087
	Urban	0.0005	-0.0016	-0.0016	-0.0043	-0.0007

^a^Family population: Total number of people living in the family; ^b^Time: Time to the nearest medical service institution; ^c^Expenditure: Family medical consumption expenditure; ^d^Average age: Average age of older couples in the family

### Relationship between differences in personal characteristics and SHOA based on CCD

**Fig. 2:**
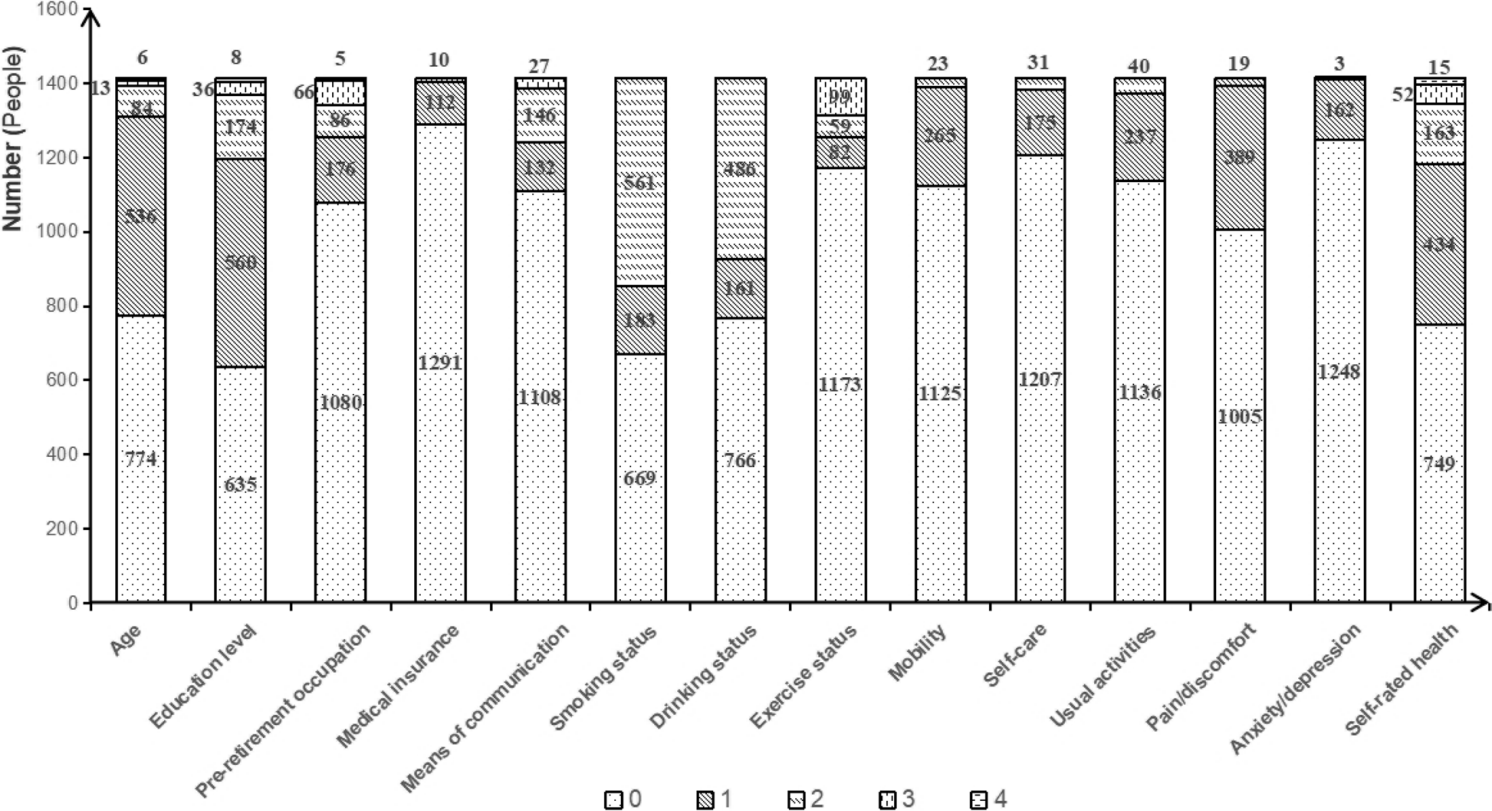
The differences in self-rated health and personal characteristics among family members 0, 1, 2, 3, 4 indicate the level difference of self-rated health status or a certain personal characteristic between older couples in the family

Figure 2 depicts the distribution (frequency) of differences between family members in terms of personal demographic background, health habits, and self-rated health status. This study also examined the association between differences in personal characteristics among family members and differences in SHOA from an intra-family perspective. The analysis found that the CCD between personal characteristics’ differences and SHOA’ differences was greater than 0.7132, and the correlation and mutual promotion were substantial. The CCD value for the difference between family members’ pre-retirement occupations and SHOA was found to be 0.9323. Likewise, the CCD value for the difference between medical insurance and SHOA was 0.9534. The analysis results of urban and rural areas show that the difference in CCD between the difference in smoking status and SHOA in urban and rural areas was relatively the largest (CCD = 0.6540, 0.8356), and the CCD based on pre-retirement occupation difference was almost equal (CCD = 0.9178, 0.9127). Except for pain/discomfort, the CCD between the differences in individual characteristics among family members and the differences in SHOA in specific dimensions was mostly greater than the overall level. See Table 7.

**Table 7:** The CCD^a^ between the “differences in self-rated health” and “differences in personal characteristics” among family members

Variable	Age^b^	Education level^b^	Pre-retirement occupation^b,d^	Medical insurance^b,e^	Means of commun-icationf^b,f^	Smoking status^b^	Drinking status^b^	Exercise status^b^
**Total**								
** Self-rated health**^**c**^	0.9225	0.9182	0.9323	0.9534	0.9208	0.7132	0.7575	0.8558
Mobility^**c**^	0.9347	0.9301	0.9463	0.9646	0.9416	0.7063	0.7506	0.9487
Self-care^**c**^	0.9404	0.9357	0.9525	0.9691	0.9505	0.7020	0.7462	0.9767
Usual activities^**c**^	0.9332	0.9279	0.9465	0.9672	0.9405	0.7122	0.7566	0.9631
Pain/discomfort^**c**^	0.9272	0.9223	0.9388	0.9603	0.9291	0.7142	0.7585	0.8448
Anxiety/depression^**c**^	0.9504	0.9464	0.9609	0.9731	0.9620	0.6836	0.7269	0.9833
**Rural**								
** Self-rated health**	0.9115	0.9044	0.9178	0.9156	0.9093	0.6540	0.7381	0.9316
Mobility	0.9350	0.9254	0.9456	0.9453	0.9219	0.6497	0.7356	0.9458
Self-care	0.9436	0.9336	0.9552	0.9556	0.9268	0.6497	0.7361	0.9504
Usual activities	0.9329	0.9250	0.9411	0.9405	0.9221	0.6551	0.7405	0.9435
Pain/discomfort	0.9077	0.9019	0.9123	0.9101	0.9072	0.6586	0.7420	0.9272
Anxiety/depression	0.9460	0.9330	0.9622	0.9636	0.9254	0.6428	0.7298	0.9526
**Urban**								
** Self-rated health**	0.9206	0.9176	0.9127	0.9364	0.9406	0.8356	0.8353	0.9221
Mobility	0.9424	0.9385	0.9281	0.9563	0.9603	0.8398	0.8391	0.9321
Self-care	0.9491	0.9444	0.9311	0.9646	0.9692	0.8378	0.8371	0.9343
Usual activities	0.9437	0.9400	0.9296	0.9567	0.9605	0.8414	0.8406	0.9331
Pain/discomfort	0.9371	0.9327	0.9226	0.9541	0.9589	0.8353	0.8350	0.9290
Anxiety/depression	0.9566	0.9516	0.9366	0.9712	0.9757	0.8394	0.8386	0.9379

^a^*CCD* Coupling coordination degree; ^b^: Represents differences in age, education level, pre-retirement occupation, medical insurance, means of communication, smoking status, drinking status, exercise status between older couples within the family; ^c^: Represents the differences in self-rated health, mobility, self-care, usual activities, pain/discomfort, anxiety/depression between older couples within the family; ^d^Pre-retirement occupation: Occupations in which older individuals spend the majority of their time prior to retirement; ^e^Medical insurance: The main type of medical insurance that older adults possess; ^f^: Indicates the communication tools and methods that older adults mainly use in their daily life

## Discussion

The purpose of this study was to evaluate the status and equity level of SHOAFL through the analysis of the health status survey data of older adults in central and southern China, and to explore the specific nature and scope of related influencing factors. The research found that the SHOAFL had a mean of 66.36 ± 15.47 and that its distribution was equitable (with a Concentration index ranging from -0.0315 to 0.0560). The SHOAFL was associated with a number of variables, including the total number of permanent family residents, the distance to the nearest medical service institution, the annual family income, the annual family medical and health expenditures, and the average age of study participants (*P* < 0.05). The results of CCD analysis showed that the differences in SHOA within the family were mainly related to the differences between individuals in medical insurance and pre-retirement occupation (*CCD* = 0.9534, 0.9232).

The total score of SHOAFL in China was 66.36 ± 15.47, which was consistent with other studies [13]. In addition to aging caused by age, the decline of older adults in terms of economic level, medical service utilization, and living environment range also affects SHOAFL [15]. At the same time, the evaluation results of urban and rural areas show that the SHOAFL in urban areas was much higher than that in rural areas. Tao HW [41] believed that the contribution rate of lifestyle to the difference in self-rated health of urban and rural older adults was 32.60%, which may be related to the lack of health knowledge acquired by older adults in rural areas and the failure to develop good living habits. On the other hand, among the various evaluation dimensions, the evaluation of self-care ability was relatively the highest, and the score of pain/discomfort was only 2.59 ± 0.46. Older persons have intermittent body pain as their physical function declines, and the human body becomes more sensitive to pain. Self-care ability is related to the quality of life and dignity of older adults, and its decay rate is slower than that of mobility and daily activities [42].

The results also indicate that the total number of people living in the family, the annual yearly family medical and health expenditure, and the average age of older adults had a significant impact on SHOAFL. The SHOAFL of families with a total resident population of 2, 3, 4, and 5 was 5.379, 4.925, 3.588, and 4.003 times that of families with a resident population of 6 or more, respectively. A study by Chen L et al. showed similar findings [43]. Alternatively, while the low average age of older families is favorable for SHOAFL, this effect will diminish as the average age rises. This may be due to the fact that some older persons with poor health failed to reach the age of 75, and a partial survivor effect was observed [44]. The current study also found that the SHOAFL of families whose nearest medical service institution was a community health service station/village clinic/outpatient department was significantly lower than that of other families. One possible explanation is that, on average, developing countries have lower overall levels of primary health care.

Furthermore, this analysis found that SHOAFL distribution was reasonable, but its beneficiaries were more likely to be from high-income families. Meanwhile, compared with rural families, this trend is more pronounced within urban families, but its individual trends were smaller than the overall trend. This finding is similar to the study by Badland H et al. [45]. This established a positive relationship between family-level income and SHOA [46]. One possible explanation is that due to economic constraints, the older adults in rural China have limited access to and quality of healthcare services. In addition, a bias was found in the distribution of SHOAFL in time from home to a medical service institution. This may be because the treatment and prognosis of some diseases are closely related to the timeliness of receiving treatment, and the time to a medical service institution has a significant impact on the timeliness of the older adults' consultation [21]. Given that family's income is a characteristic that is difficult to change quickly, policymakers and organizers should prioritize minimizing the time spent travel to medical service providers for older adult families, especially rural families.

Finally, differences in SHOA within the family were strongly correlated and mutually reinforcing with differences in personal characteristics among family members, but were mainly attributable to differences in medical insurance and pre-retirement occupations between husbands and wives. This reminds those external characteristics such as economic status and living habits also play a role in the differences in the health of couples within the family. Therefore, the authors suggest that when the government formulates older adult care service policies and provides corresponding services in the community, they could pay more attention to the health differences between older adults within the family due to different economic status, rather than simply carrying out education and assistance in the family unit.

This study has several strengths: Firstly, the authors focused their study direction on SHOAFL. Secondly, the study analyzed the equity of the distribution of SHOAFL using the concentration index. Thirdly, the evaluation results based on CCD can give evidence-based references for understanding and enhancing disparities in self-rated health among older adult couples. However, a significant limitation of this study is that the family-based design fails to include the daily care of children, spiritual comfort and other support factors within the family in the study, and fails to consider the health utility value of older adults. In addition, methods such as CCD used in this study have strict requirements on the quantity and quality of data, resulting in dichotomous physical health factors such as chronic disease not being included in the study in the last part of the results, and limiting their applicability to other similar investigations. Thirdly, given the cross-sectional nature of the current study, the authors believe that there may be other important long-term changing factors, such as socioeconomic development and conceptual changes, which merit further exploration and analysis.

## Conclusions

This study assessed the current status and equity levels of SHOAFL in central and southern China, and explored factors associated with SHOAFL’s status and equity. The results show that SHOAFL was generally, and they were more inclined to urban families with high income and short time to medical service institution. The observed differences in SHOA within families were mainly related to differences between individuals in health insurance and pre-retirement occupations. Policymakers could increase the equity of SHOAFL by making services more accessible to rural residents with low incomes. At the same time, narrowing the disparity in health insurance between older couples could also help to improve their health status.

## Data Availability

Availability of data supporting the findings of this study is limited and therefore not publicly available. Data are however available from the corresponding author upon reasonable request.

## Abbreviations

SHOA: Self-related health of older adults
SHOAFL: Self-related health of older adults at the family level
LR: Logistic regression
CCD: Coupling Coordination Degree
EQ-5D-3L: Three-level European five-dimensional health scale
